# Complexities and Perplexities: A Critical Appraisal of the Evidence for Soil-Transmitted Helminth Infection-Related Morbidity

**DOI:** 10.1371/journal.pntd.0004566

**Published:** 2016-05-19

**Authors:** Suzy J. Campbell, Susana V. Nery, Suhail A. Doi, Darren J. Gray, Ricardo J. Soares Magalhães, James S. McCarthy, Rebecca J. Traub, Ross M. Andrews, Archie C. A. Clements

**Affiliations:** 1 Research School of Population Health, College of Medicine, Biology, and Environment, The Australian National University, Canberra, Australian Captial Territory, Australia; 2 Molecular Parasitology Laboratory, QIMR Berghofer Medical Research Institute, Brisbane, Queensland, Australia; 3 Children's Health and Environment Program, Queensland Children's Medical Research Institute, The University of Queensland, Brisbane, Queensland, Australia; 4 School of Veterinary Science, The University of Queensland, Gatton, Queensland, Australia; 5 School of Population Health, University of Queensland, Brisbane, Queensland, Australia; 6 Clinical Tropical Medicine Laboratory, QIMR Berghofer Medical Research Institute, Brisbane, Queensland, Australia; 7 Faculty of Veterinary and Agricultural Science, The University of Melbourne, Melbourne, Victoria, Australia; 8 Menzies School of Health Research, Charles Darwin University, Darwin, Northern Territory, Australia; Swiss Tropical and Public Health Institute, SWITZERLAND

## Abstract

Background: Soil-transmitted helminths (STH) have acute and chronic manifestations, and can result in lifetime morbidity. Disease burden is difficult to quantify, yet quantitative evidence is required to justify large-scale deworming programmes. A recent Cochrane systematic review, which influences Global Burden of Disease (GBD) estimates for STH, has again called into question the evidence for deworming benefit on morbidity due to STH. In this narrative review, we investigate in detail what the shortfalls in evidence are. Methodology/Principal Findings: We systematically reviewed recent literature that used direct measures to investigate morbidity from STH and we critically appraised systematic reviews, particularly the most recent Cochrane systematic review investigating deworming impact on morbidity. We included six systematic reviews and meta-analyses, 36 literature reviews, 44 experimental or observational studies, and five case series. We highlight where evidence is insufficient and where research needs to be directed to strengthen morbidity evidence, ideally to prove benefits of deworming. Conclusions/Significance: Overall, the Cochrane systematic review and recent studies indicate major shortfalls in evidence for direct morbidity. However, it is questionable whether the systematic review methodology should be applied to STH due to heterogeneity of the prevalence of different species in each setting. Urgent investment in studies powered to detect direct morbidity effects due to STH is required.

## Introduction

Soil-transmitted helminth (STH) infections are among the most prevalent of the neglected tropical diseases (NTDs), characterised by chronic and subtle impacts on human health and development. They rarely cause direct mortality; instead they are major contributors to morbidity. Morbidity effects of STH infection are difficult to quantify given the long duration of infection, often over many years, presence of other concurrent diseases, and factors such as poverty and malnutrition, to which they are strongly linked, and which can confound measures of STH-associated morbidity. Accurate quantification of STH-associated morbidity and disease burden is critical to rationalise large-scale deworming programmes.

Debate exists around the health benefits and cost-effectiveness of STH intervention strategies, rising to prominence with Cochrane systematic reviews reporting equivocal evidence of health benefits [1–4] and a statistical re-analysis of a major deworming trial from 1998–1999 that found differing results to original author conclusions [5,6]. In response, concerns have been raised about methodological bias in systematic reviews [7], the importance of not confining anthelmintic treatment to infected children [7,8], the economic importance of deworming [9], and the conclusions drawn in the replication analyses [9,10]. The Cochrane systematic review, the replication analyses, and the resultant discussions are positive insofar as they will further progress an evidence-enhancing research agenda, but there is a strong underlying imperative not to adversely influence international policy for mass deworming.

Global burden of disease (GBD) studies quantifying STH burden, with the most recent estimates published in 2012 [11], have also been subject to debate, in part due to the exclusion of certain morbidities because available evidence is deemed insufficient to justify inclusion. The findings from Cochrane systematic reviews influence what morbidities are included in GBD estimates, which, also, can be used to influence policy; therefore, it is important to understand what the underlying shortfalls are.

It is timely to revisit the pool of knowledge for STH impact on health, with a particular focus on how recent evidence contributes to knowledge gaps, and to critically appraise the approaches being used in systematic reviews, particularly the Cochrane systematic review, to disentangle why results are consistently inconclusive.

In this narrative review, we critically appraise narrative and systematic reviews and consider the recent observational and experimental literature, covering both STH morbidity and treatment associations with health outcomes. We explore the reasons for the reported lack of effect in the most recent Cochrane systematic review and investigate why conclusions regarding STH impact on haemoglobin do not concur with other systematic reviews [12,13]. This paper is not a meta-analytic paper (which would replicate previous reviews). By considering both well-known and more recent evidence, we provide an updated perspective of where evidence is insufficient to enable conclusions on STH morbidity to be drawn and highlight where research needs to be directed in future.

## Methods

We searched scientific literature in the MEDLINE database (January 2000 to January 2016) for evidence of any morbidity or mortality outcomes associated with *Ascaris lumbricoides*, *Trichuris trichiura*, and the hookworms *Necator americanus*, *Ancylostoma duodenale*, and *Ancylostoma ceylanicum*. The nematode *Strongyloides stercoralis* was not included in this review. Specifically, the following combination of text and Medical Subject Headings (MeSH) terms was used: (“*Necator americanus*” or “*Ancylostoma duodenale*” or “*Ascaris lumbricoides*” or “*Trichuris trichiura*” or “*Ancylostoma ceylanicum*” or hookworm or “soil-transmitted helminth”) and (morbidity or mortality or anaemia or stunting or retardation or wasting or malnutrition or cognitive or cognition or impair). The search was limited by English language. This search aimed to (1) identify narrative and systematic reviews of STH morbidity and (2) identify recent research papers on STH-associated morbidity. Article abstracts were reviewed and literature was retrieved if there was specific reference to a morbidity or mortality outcome from STH or if they could not be excluded (i.e., if the abstract did not clearly indicate morbidity outcomes). Reference lists of identified articles were cross-checked. We further cross-checked peer-reviewed literature with information from United Nations, World Health Organization (WHO), and several other non-government organisation websites.

Once identified, narrative and systematic reviews were analysed to determine current knowledge and evidence gaps (Tables 1 and 2). Critical appraisal checklists were used to analyse systematic reviews [14]. For the systematic reviews, we focussed on the research question being investigated (null hypothesis), search and selection criteria, trials selected, inclusion or exclusion of factors such as concurrent diseases or interventions, definitions given by authors to “quasi” randomised controlled trials (RCTs), evidence rankings from authors, definition of trial participants, baseline measures, classes of infection intensity, intervention and outcome measures, consideration of absolute versus relative outcome measures, length of follow-up, pooling of results and sub-group analyses undertaken, and the conclusions drawn within manuscripts. We did not analyse bias or adjusting of intra-class correlations in cluster RCTs, but we did check that the systematic reviews had investigated these. We did not investigate the specific meta-regression methods of the models. Given the requirement for regular updates of Cochrane systematic reviews, we only included the most recent of these. We report our findings in relation to the most recent Cochrane systematic review.

**Table 1:** Summary of existing evidence from narrative reviews of soil-transmitted helminth morbidity published since 2000.

**Topic area:** STH entry and establishment*	
**Citation**	**Reported evidence**
O’Lorcain and Holland, 2000 [15]; Crompton, 2001 [16]; Bethony et al., 2006 [17]; Albonico et al., 2008 [18]; Brooker, 2010 [19]; Periago and Bethony, 2012 [20]; Craig and Scott, 2014 [21]; Ojha et al., 2014 [22]	Direct oral infection of *A*. *duodenale* can cause Wakana disease, characterized by nausea, fever, vomiting, pharyngeal irritation, cough, shortness of breath, and hoarseness. Penetration of hookworm larvae into the skin causes intense itching and often a cutaneous rash. *A*. *lumbricoides* and hookworms migrate through the vascular system and respiratory tract before being coughed from the lungs and swallowed, with accompanying clinical symptoms including (for hookworms) mild cough resembling Löffler’s syndrome, sore throat and fever, and (for *A*. *lumbricoides*) *Ascaris* pneumonia and hypersensitivity, occasionally manifesting as asthma. Larval migration to the gastrointestinal tract causes symptoms including rising peripheral eosinophilia, moderate to severe epigastric pain, intense nausea, exertional shortness of breath, pain in the lower extremities, palpitations, abdominal tenderness, joint and sternal pain, headache, fatigue, impotence, flatulence, weight loss, and/or moderately severe diarrhoea. Infections cause clearly discernible intestinal damage. Deworming children has been shown to clearly reduce intensity of helminth burden and associated symptoms.
**Topic area:** Blood loss and anaemia	
**Citation**	**Reported evidence**
Crompton, 2000 [23]; Guyatt, 2000 [24]; O’Lorcain and Holland, 2000 [15]; Drake and Bundy, 2001 [25]; Stephenson, 2001 [26]; WHO, 2002 [27]; De Silva, 2003 [28]; Brooker et al., 2004 [29]; Hotez et al., 2004 [30]; Savioli et al., 2004 [31]; Bethony et al., 2006 [17]; Hotez et al., 2006 [32]; Larocque and Gyorkos, 2006 [33]; Albonico et al., 2008 [18]; Brooker, 2010 [19]; Elliott et al., 2011 [34]; Tchuem Tchuenté, 2011 [35]; Hall et al., 2012 [36]; Periago and Bethony, 2012 [20]; Ojha et al., 2014 [22]	Hookworms cause intestinal blood loss leading to iron deficiency (including iron deficiency anaemia [IDA]) and protein malnutrition. Effects worsen with increased intensity of infection. Anaemia, in turn, is strongly linked to poor underlying iron status, malaria, poverty, and other factors. *T*. *trichiura* is associated with blood loss, chronic inflammation, iron deficiency, and protein loss in children. Blood loss from *T*. *trichiura* infection is likely to contribute to anaemia, particularly if hookworms are also harboured or the individual has low dietary iron intake. No evidence that *A*. *lumbricoides* infection leads to iron malabsorption and IDA in children. IDA during pregnancy is linked to severe maternal anaemia. Both IDA and severe anaemia in pregnancy contribute to poor maternal health, maternal mortality, premature delivery, low infant birthweight, and impaired lactation. *A*. *duodenale* infection during pregnancy may be lactogenically transmitted to neonates. Deworming children clearly shows improvements in iron status and reduced chance of developing IDA and severe anaemia. Deworming will reduce helminth infections, but nutritional interventions are required to restore health. Some (but not all) studies show that deworming pregnant women, with or without iron folate supplementation, can improve maternal haemoglobin (Hb) levels, reduce severe anaemia and stillbirths, and improve infant birthweight and survival. Albendazole treatment during pregnancy may increase risk of eczema in the population.
**Topic area:** Physical development, fitness, worker productivity	
**Citation**	**Reported evidence**
Crompton, 2000 [23]; Guyatt, 2000 [24]; O’Lorcain and Holland, 2000 [15]; Stephenson et al., 2000 [37]; Stephenson, 2001 [26]; WHO, 2002 [27]; De Silva, 2003 [28], Brooker et al., 2004 [29]; Hotez et al., 2004 [30]; Savioli et al., 2004 [31]; Bethony et al., 2006 [17]; Hotez et al., 2006 [32]; Hall, 2007 [38]; Albonico et al., 2008 [18]; Brooker, 2010 [19]; Tchuem Tchuenté, 2011 [35]	Chronic hookworm, *A*. *lumbricoides*, and *T*. *trichiura* infections associated with reduced weight, height, skinfold thickness/arm circumference, and appetite in children, with effects more pronounced in heavy infections. Even light infections are considered likely to contribute to growth deficits if underlying nutrition is poor. Severe stunting can occur in *Trichuris* Dysentery Syndrome (TDS). *A*. *lumbricoides* associated with lower vitamin A absorption and lactose intolerance in children. Other physical effects of hookworm include dermatitis, anasarca, oedema of face and limbs, potbelly, waxy skin, and worm passing. Most of these effects may be attributable to iron deficiency. Many (but not all) RCTs found improvements in height, weight, arm circumference, appetite, and physical activity (step tests) after deworming schoolchildren and younger children. STH is associated with poor economic productivity, but direct evidence is lacking. Difficult to quantify, as iron deficiency and IDA are characterized by weakness and fatigue in adults and some studies have linked IDA (measured Hb level) to productivity. An economic analysis estimated that curing hookworm infection in United States of America led to 25% increased likelihood of children attending school than untreated children, translating into 45% higher earnings by 1940.
**Topic area:** Cognitive development	
**Citation**	**Reported evidence**
Crompton, 2000 [23]; Guyatt, 2000 [24]; O’Lorcain and Holland, 2000 [15]; Stephenson et al., 2000 [37]; Crompton, 2001 [16]; Drake and Bundy, 2001 [25]; Stephenson, 2001 [26]; WHO, 2002 [27]; De Silva, 2003 [28]; Brooker et al., 2004 [29]; Hotez et al., 2004 [30]; Savioli et al., 2004 [31]; Bethony et al., 2006 [17]; Hall, 2007 [38]; Albonico et al., 2008 [18]; Brooker, 2010 [19]; Tchuem Tchuenté, 2011 [35]	Strong hookworm associations with intellectual growth retardation, cognitive performance in school, reduced school attendance, and educational deficits; however, evidence is not yet considered causal. Most of these effects may be attributable to iron deficiency. There are agreed strong associations between iron deficiency and cognitive performance. TDS also associated with a marked decrease in cognitive score tests. Regular deworming of schoolchildren has shown improvements in some measures of cognitive performance, educational achievement, and school attendance.
**Topic area:** Multiparasitism and other disease interactions**	
**Citation**	**Reported evidence**
Drake and Bundy, 2001 [25]; Brooker et al., 2004 [29]; Bethony et al., 2006 [17]; Borkow and Bentwich, 2006 [39]; Mwangi et al., 2006 [40]; Albonico et al., 2008 [18]; Geiger, 2008 [41]; Hotez et al., 2008 [42]; Pullan and Brooker, 2008 [43]; Steinmann et al., 2010 [44]; Elliott et al., 2011 [34]; Gerns et al., 2012 [45]; Webb et al., 2012 [46]	Helminth species, including *Schistosoma mansoni*, are often comorbid in humans. Individuals harbouring multiple helminth species often harbour higher-intensity infections from each species than those with single-species infections; this may lead to additive impacts on morbidity as infection intensity is closely linked to severity of disease. STH are often co-endemic with malaria and Human Immunodeficiency Virus (HIV) and may increase host susceptibility to these and possibly other infectious diseases such as tuberculosis. STH and malaria in particular are known aetiological contributors to anaemia; the extent to which they may interact and potentially worsen anaemia is unknown. *A*. *lumbricoides* may reduce post-vaccination immune responses to tetanus vaccine and STH infection has been suspected to lower the efficacy of Bacille Calmette—Guerin (BCG) vaccine. Some studies have found no effect of anthelmintics on response to immunisation.
**Topic area:** Additional *A*. *lumbricoides* morbidity	
**Citation**	**Reported evidence**
O’Lorcain and Holland, 2000 [15]; Crompton, 2001 [16]; Savioli et al., 2004 [31]; Albonico et al., 2008 [18]; Brooker, 2010 [19]; Hesse et al., 2012 [47]; Ojha et al., 2014 [22]	Intestinal obstruction due to *A*. *lumbricoides* is regularly encountered in children from developing countries. Children tend to harbour more *A*. *lumbricoides* than adults. Complications of the biliary system occur in adults. Both of these conditions can be fatal. Numerous ectopic sites for *A*. *lumbricoides* migration have been encountered, often with risks, e.g., airway obstruction. Mortality rates from ascariasis are difficult to estimate. Deworming has been clearly shown to reduce the frequency of complications. However, intestinal blockage arising from anthelmintic purging of *A*. *lumbricoides* has been reported.
**Topic area:** Additional *T*. *trichiura* morbidity	
**Citation**	**Reported evidence**
Stephenson et al., 2000 [48]; Drake and Bundy, 2001 [25]; Savioli et al., 2004 [31]; Albonico et al., 2008 [18]; Brooker, 2010 [19]; Wang et al., 2013 [49]; Ojha et al., 2014 [22]	TDS associated with heavy *T*. *trichiura* infection mainly occurs in children and leads to severe growth stunting and cognitive deficits that might not be reversible. Other TDS symptoms include chronic dysentery, rectal prolapse, anaemia, and clubbing of fingers. TDS can be fatal. Lighter *T*. *trichiura* infections are also problematic, especially in children.
**Topic area:** *A*. *ceylanicum*	
**Citation**	**Reported evidence**
Traub, 2013 [50]	*A*. *ceylanicum* may have important morbidity, but sparse evidence is available. In experimental infections, *A*. *ceylanicum* can cause “ground itch” and moderate to severe abdominal pain, diarrhoea, and occult blood in the faeces accompanied by peripheral eosinophilia. Patent *A*. *ceylanicum* can produce chronic infections that may occur in high enough burdens to produce anaemia.

* This evidence is grouped by topic area as many narrative reviews referred to the same primary evidence.

** Not being considered further in this review.

**Table 2:** Evidence from systematic reviews of soil-transmitted helminth morbidity published since 2000 (selected according to specified criteria, arranged by date of most to least recent).

Citation	Included studies and focus areas	Reported results	Comments
Taylor-Robinson et al., 2015 [4] Previous versions: Dickson et al., 2000 [1]; Taylor-Robinson et al., 2007 [2], 2012 [3].	RCTs and quasi-RCTs, comparing deworming in any of the four STH species with placebo/no treatment in children aged 16 years or less, reporting on weight, haemoglobin (Hb), intellectual development, school attendance, school performance, and mortality.	Treating infected children with a single dose of deworming drugs may increase weight gain over 1–6 months. There is insufficient evidence to know whether treatment of known infected children has effects on Hb, school attendance, cognitive functioning, or physical well-being. Treating all children living in endemic areas with a single dose of deworming drugs probably has little or no effect on average weight gain, average Hb, or average cognition. Regularly treating all children in endemic areas with deworming drugs, given every 3–6 months, may have little or no effect on average weight gain, average height, average Hb, formal tests of cognition, exam performance, or mortality. Very limited evidence to assess an effect on school attendance. Insufficient evidence to do subgroup analysis by age.	See main text for detailed comments. Study selection criteria included all STH species and all anthelmintics included in the WHO Model List of Essential Medicines. They only included studies with other interventions (e.g., micronutrients) in which these interventions were given to the intervention and control arms. They only included studies of children. They considered 37 (of 45 total) trials for which no baseline screening of STH prevalence was done, although studies were from endemic areas. Observational evidence not included on the basis of reducing residual confounding. Some of the conclusions differ from those of Smith and Brooker, 2010 and Gulani et al., 2007.
Yap et al., 2014 [51]	RCTs and prospective cohort studies assessing nutrition influence, with and without anthelminthic drugs, on STH infection and reinfection.	Positive effects of nutritional supplementation or the host’s natural nutritional status on (re-)infection with STH were reported in 14 studies, while negative effects were documented in six studies. Multi-micronutrients did not significantly impact on STH re-infection rates. Current evidence for effect of nutrition on (re-)infection with STH is weak and of low quality.	Studies were investigated by different STH.
Smith and Brooker, 2010 [12]	Observational and experimental evidence investigating hookworm impact on anaemia (non-pregnant populations). Compared Hb concentration between uninfected and hookworm-infected individuals (of different intensities). Meta-analysis of RCTs to investigate deworming impact on Hb and anaemia.	Observational studies: moderate- and heavy-intensity hookworm infections associated with lower Hb in school-aged children. All infection intensities associated with lower Hb in adults. Intervention studies: albendazole corresponded to increased mean Hb; mebendazole had no impact. Greatest mean Hb increase when albendazole co-administered with praziquantel. No measured benefit of benzimidazoles with iron supplementation. For anaemia, benzimidazoles alone had small impact on moderate anaemia. Larger impact on anaemia from benzimidazoles with praziquantel.	Study selection criteria included hookworms only, benzimidazole treatments (alone, and with praziquantel or iron supplementation) from 1980 onwards, and required baseline hookworm assessment for inclusion. Considered effects of different treatment types: albendazole alone, mebendazole alone, albendazole plus praziquantel, albendazole plus iron supplementation. Could not differentiate by hookworm species. Some of the conclusions differ from those of Taylor-Robinson et al., 2015.
Haider et al., 2009 [52]	Three RCTs investigating effect of administration of anthelmintics during the second or third trimester on maternal and child health outcomes.	Single dose of anthelmintic in second trimester of pregnancy had no associated impact on maternal anaemia in the third trimester. Single dose of anthelmintic plus iron supplementation in the second and third trimester of pregnancy had no associated impact on maternal anaemia in the third trimester compared to iron supplementation alone. No impact was found for low birthweight, perinatal mortality, or preterm birth. Impact on infant survival at six months of age not evaluated. Current evidence insufficient to recommend use of anthelmintic for pregnant women after the first trimester of pregnancy.	Assessed the same two RCTs as Brooker et al., 2008, with inclusion of a third RCT. Whilst Haider et al., 2009 undertook meta-analysis, they have the same conclusions as Brooker et al., 2008; that evidence is currently insufficient. More RCTs of pregnant women should be undertaken to strengthen evidence.
Brooker et al., 2008 [53]	Observational and experimental evidence investigating hookworm impact on Hb concentration in pregnant women. Compared Hb concentration between uninfected and lightly-infected women, and between lightly-infected and heavily-infected women.	Observational studies: increasing hookworm infection intensity was statistically associated with lower Hb levels in pregnant women in poor countries. Intervention studies: two RCTs identified; other evidence also included. Could not quantify benefit of anthelmintics in RCTs due to different outcome measures. RCTs showed deworming benefit on maternal or child health. Concluded that there are insufficient data to quantify the benefits of deworming.	More RCTs of pregnant women should be undertaken to strengthen evidence.
Gulani et al., 2007 [13]	RCTs and quasi-RCTs assessing routine deworming impact on Hb, in any population.	Ten of the studies used albendazole as the anthelmintic drug, three used mebendazole, and one used bephenium. Routine administration of anthelmintics results in a marginal increase in Hb.	This review did not distinguish between different STH species or account for intensity of infection, which may have underestimated true treatment effect [12,54]. Some of the conclusions differ from those of Taylor-Robinson et al., 2015.

**Fig 1:**
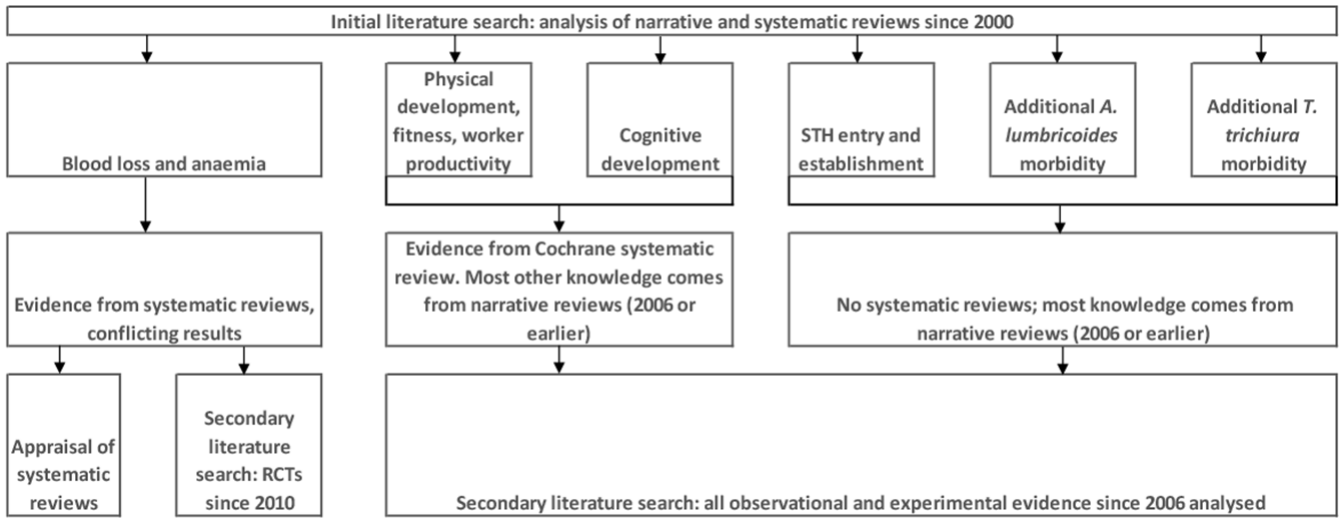
Literature selection flow diagram.

We further investigated recent evidence to determine whether knowledge gaps identified in earlier reviews were being addressed. Informed by the evidence summaries (Tables 1 and 2), we repeated the literature search (Fig 1, Table 3), applying the following criteria:

(i) If studies related to hookworm impact on haemoglobin or anaemia, we included only experimental evidence since the 2010 systematic review was done;
(ii) If studies related to impact on haemoglobin or anaemia of other STH, or any STH impact on physical (e.g., stunting or wasting) or cognitive measures of morbidity, we included studies from 2006 (the date of the last major STH review) and considered all observational and experimental evidence, with the exception of case series and case reports; and
(iii) In the absence of any identified epidemiological investigations into *A*. *lumbricoides* or *T*. *trichiura* acute complications, we included five case series, but not individual case reports.

**Table 3:** Observational and experimental contributions to soil-transmitted helminth morbidity; evidence since 2006.

Citation and country	Sample size and population	Study design and description	Results	Comments
Haemoglobin and anaemia*				
Gyorkos et al., 2011, 2012 [55,56], Peru	935 pregnant women (474 intervention, 461 control)	Double-blind RCT; mebendazole plus iron supplements (intervention), placebo plus iron supplements (control). Assessed impact of mebendazole on maternal STH intensity and prevalence. Recruited second trimester; followed up at delivery. Randomisation reported in [57]: computer-generated allocation. STH diagnosed by Kato-Katz.	Recruited 1,042 women; 935 (89.7%) had complete information for analyses. Baseline (second trimester) prevalence of hookworm 46.3%, *A*. *lumbricoides* 63.9% and *T*. *trichiura* 82.0%. Women in highest two *T*. *trichiura* intensity quintiles had significantly lower mean Hb concentrations than lowest quintile. Higher baseline intensities of hookworm and *T*. *trichiura* infections associated with higher risk of anaemia in third trimester. Overall, women with moderate ⁄ heavy *T*. *trichiura* infection were at higher risk of anaemia; highest risk among those with moderate ⁄ heavy hookworm co-infection. Mebendazole significantly reduced prevalence and intensity of all STH infections, but not anaemia risk.	Data originated from an RCT investigating the effect of mebendazole on birth weight [33]; these data assess different trial outcomes.
Ndyomugyenyi et al., 2008 [58], Uganda	832 pregnant women with confirmed STH infection at baseline (198 ivermectin, 194 albendazole, 199 ivermectin plus albendazole, 241 uninfected women [reference])	Open-label RCT; investigating efficacy and recorded adverse events among STH-infected women of ivermectin only, albendazole only, and ivermectin plus albendazole against STH infections on maternal health and neonatal outcomes. Reference group of uninfected women (non-randomised). STH diagnosed by Kato-Katz. Recruited second trimester; followed up at delivery.	832 women provided baseline stool; 636 (76.4%) followed up. Baseline STH prevalence 71% (hookworm 66.6%, *T*. *trichiura* 4.6%, *A*. *lumbricoides* <0.1%). Other parasite prevalence: 35.2% *Plasmodium falciparum*; 3.7% *Schistosoma mansoni*. Maternal anaemia prevalence reduced in all groups with no significant between-group difference at 36 weeks. Significant difference in mean Hb levels between first antenatal care visit and at 36 weeks of gestation for most (but not all) treatment groups and also reference group. Significant difference in mean Hb levels at 36 weeks between some (but not all) treatment groups and reference group. No significant difference in infant anaemia between treatment and reference groups. Mean infant Hb at birth not significantly different among groups, although significant difference was noted between one treatment group and reference group. No significant difference for mean birth weight, frequency of low birth weight, premature birth, stillbirth, or neonatal mortality.	Infection status determined pre-randomisation, but randomisation method not detailed. Women with severe anaemia excluded, which could have resulted in lack of difference in anaemia status in trial arms at 36 weeks. Study not powered to detect rare effects (adverse pregnancy outcomes) [58].
Quihui-Cota et al., 2010 [59], Mexico	73 School-aged children (SAC) aged 6–10 years, of confirmed *T*. *trichiura* infection or no parasite infection (excluded children with other parasite infections or co-infections)	Cross-sectional (longitudinal follow-up of 5 weeks); investigated association between trichuriasis and iron status	*T*. *trichiura* prevalence 45.2%. Height for age significantly higher in the *T*. *trichiura*-uninfected than *T*. *trichiura*-infected children. Significantly higher baseline concentrations of Hb, haematocrit, blood cell count (RBC), and serum iron in *T*. *trichiura*-uninfected compared with *T*. *trichiura*-infected children. Trichuriasis and Hb associated in adjusted analyses. Hb, RBC, and serum ferritin concentrations significantly increased in infected children 5 weeks after treatment.	Not large numbers. Regression analysis adjusted for sex and age.
**Physical development, fitness, worker productivity—Preschool-aged children (PSAC)**				
Awasthi et al., 2008 [60], India	3,935 PSAC (aged 1–5 at baseline) (1852 intervention, 1860 controls for analyses)	Open-label cluster RCT of 50 urban slums; 25 received albendazole plus usual care (intervention), 25 received usual care only (control). Computer-generated random allocation. Investigated impact of five rounds of anthelmintic treatment administered every 6 months over 2 years on PSAC height and weight.	Baseline recruitment 3,935 children; 3,712 (94.3%) followed up. Community-based programme; no baseline STH assessment done. At final follow up, albendazole-treated arm had similar height gain but a 35% greater weight gain, equivalent to an extra 1 kg over 2 years.	2-year follow-up carefully considered in power calculations for ability to detect treatment effect in a generally malnourished population. No measure of egg counts from STH (but treatment effects are attributable to deworming intervention in this trial design).
Joseph et al., 2014, 2015 [61,62], Peru	1,760 PSAC (12 and 13 month olds) in a deworming trial (NB: baseline analyses are for subgroup of 880 children with stool analysed by Kato Katz)	Double-blind RCT, investigating deworming impact on growth in one-year-old children. Children enrolled at 12 month clinic visit, followed up at 18 and 24 month visits. Computer-generated random allocation to: (i) deworming at 12 months and placebo at 18 months; (ii) placebo at 12 months and deworming at 18 months; (iii) deworming at both 12 and 18 months; (iv) placebo at both 12 and 18 months (control group). Cross-sectional baseline survey (nested) conducted to assess association between malnutrition (i.e., stunting and underweight) and STH infection. Primary RCT outcome: weight gain at 24 month visit.	Baseline recruitment 1,760 children; follow-up of 1,563 children (88.8%). STH prevalence 14.5% (*A*. *lumbricoides* 11.5%, *T*. *trichiura* 4.5%, hookworm 0.6%). Risk factors for stunting included infection with at least one STH species and a lower development score. No statistically significant association found between any STH infection and being underweight. At RCT follow-up, there was no statistically significant difference in weight gain in any of the deworming intervention groups compared to the control group.	Baseline survey reported in [62]. Baseline analyses adjusted for sex, age, birth weight, any hospitalizations since birth, development score, and socioeconomic status. Models run as any versus no STH infection, not by species.
LaBeaud et al., 2014 [63], Kenya	545 PSAC aged 0–3 years	Prospective maternal-child cohort followed up every 6 months for 3 years. Assessed the prevalence of parasitic infections and their association with growth in very early childhood.	STH prevalence 19% (undifferentiated) by three years of age; point prevalence of hookworm and *A*. *lumbricoides* not known. Malaria 12%, filariasis 4.8%, schistosomiasis 2.9% by age three years. Hookworm infection by 24 months associated with below average growth in length and head circumference at 24 months and relative deficit in overall weight gain over first 36 months of life. Hookworm infection by 30 months associated with a decreased z-score for head circumference at 30 months, infection by 36 months of age associated with a relative decrease in weight gained at 36 months. *A*. *lumbricoides* infection by either 12 or 18 months associated with a decrease in weight z-scores at each age milestone, respectively. *A*. *lumbricoides* infection by 24 months associated with decreased length achieved at 24 months. For 180 infants followed up at every visit through 24 months, hookworm infection by 24 months associated with decrease in weight and head circumference z-score at 24 months. *A*. *lumbricoides* infection by 24 months associated with decrease in linear growth (length z-score) at 24 months.	Analyses adjusted infant’s sex, birth weight, birth length, birth head circumference, and maternal education.
Suchdev et al., 2013 [64], Kenya	205 PSAC, mean age 3.2 ± 0.08; 487 SAC, mean age 11.4 ± 0.13	Cross-sectional; to evaluate nutritional impact of STH infection	STH prevalence 39.7% (*A*. *lumbricoides* 23.0%, *T*. *trichiura* 26.3%, hookworm <0.1%). Any STH infection, and *A*. *lumbricoides* infection significantly associated with vitamin A deficiency (VAD). Children with moderate-intensity *A*. *lumbricoides* infection more likely to have VAD than children without *A*. *lumbricoides* infection. Associations remained when models adjusted for reported history of vitamin A supplementation. High-intensity *A*. *lumbricoides* infection positively associated with anaemia. No nutritional associations significant in SAC.	Analyses adjusted for age, sex, and socioeconomic status
Casapía et al 2007 [65], Peru	252 PSAC aged <5 years	Cross-sectional; to determine prevalence of malnutrition and its risk factors	STH prevalence: *T*. *trichiura* 38.9%, *A*. *lumbricoides* 32.1%, hookworm 3.2%. Moderate- to high-intensity *T*. *trichiura* infection and any hookworm infection were risk factors for moderate to severe wasting. Moderate- to high-intensity *T*. *trichiura* infection was a risk factor for moderate to severe underweight. STH were not risk factors for moderate to severe stunting.	Analyses adjusted for child gender and maternal age
Gutierrez-Jimenez et al., 2013 [66], Mexico	250 PSAC aged <5 years from three impoverished municipalities	Cross-sectional	STH prevalence 38.8% (*A*. *lumbricoides* 33.6%, *T*. *trichiura* 1.2%, hookworm not reported). Malnutrition associated with presence of intestinal parasites in children from one village.	Unadjusted analyses; results for malnutrition not tabulated.
Gyorkos et al., 2011 [67], Peru	349 PSAC; 164 aged 7–9 months, 185 aged 12–14 months	Cross-sectional; to (1) examine prevalence patterns of helminth infection in early childhood; (2) assess association between helminth infection and socio-demographic characteristics; and (3) examine effect of intensity of helminth infection on stunting and anaemia	STH infections first appeared in children at eight months of age. In the 7–9 month age group STH prevalence was 4.3% (*A*. *lumbricoides* 1.8%, *T*. *trichiura* 1.2%, hookworm 1.2%). For the 12–14 month age group, STH prevalence was 29.2% (*T*. *trichiura* 21.2%, *A*. *lumbricoides* 12.4%, hookworm 0.5%). Among infected children, moderate-to-heavy infection of any STH was associated with stunting.	Very low numbers—seven moderate- to heavy-infected children. Analyses adjusted for age, sex, mother’s education, and intensity of STH infection
**Physical development, fitness, worker productivity—mixed PSAC and SAC**				
Hürlimann et al., 2014 [68], Côte d’Ivoire	852 individuals (anthropometry for children aged <18 years only)	Cross-sectional survey (two communities); investigated patterns of polyparasitism, including associations and interactions between infection, clinical indicators, and self-reported morbidity.	Prevalence of hookworm 32.4% and 26.8% in the two communities, respectively; *A*. *lumbricoides* and *T*. *trichiura* infections rare. Most STH infections were light intensity. Polyparasitism common. In analysis of hookworm mono-infection, infected children had significantly lower odds of anaemia.	Opposite direction of effect to anticipated. All models were adjusted by age group, sex, and socioeconomic status.
Oninla et al., 2010 [69], Nigeria	749 children aged 3–19 years	Cross-sectional; assessed STH impact on nutritional status	Baseline STH prevalence 30.0%. Hookworm infection a significant risk factor for underweight, wasting and stunting. *T*. *trichiura* a significant risk factor for stunting.	Reported as multivariable model results, but not tabulated and adjusted variables not noted.
Staudacher et al., 2014 [70], Rwanda	622 children aged 4–18 years across two sites (301 rural, 321 urban)	Pre and post (15 week follow-up), assessed prevalence, associated factors, and manifestation of STH infection, cure, and reinfection rates. Intervention: single dose mebendazole.	Baseline STH prevalence 25.4% (38.2% urban, 13.4% rural); *A*. *lumbricoides* 24.3%. STH infection associated with reduced height-for-age, stunting, any clinical finding, anaemia and low Hb levels in urban but not rural children (who exhibited worse clinical parameters). Clinically assessed malnutrition associated with STH infection only in rural children.	Analyses adjusted for factors found to be univariately significant.
Cabada et al., 2015 [71], Peru	240 children aged 3–12 years	Cross-sectional; to evaluate prevalence of soil-transmitted helminth infections, anaemia, and malnutrition	Parasite prevalence: *A*. *lumbricoides* 14.2%, *Fasciola hepatica* 9.6%, *Hymenolepsis nana* 9.3%, *T*. *trichiura* 1.3%, hookworm 1.7%, *Strongyloides stercoralis* 0.8%, *Giardia intestinalis* 27.5%. Stunting and wasting not associated with helminth infections.	Low STH prevalence. Analyses adjusted for socioeconomic and demographic variables, and variables P≤ 0.1 in bivariate analysis. Regression results not tabulated.
Sekiyama et al., 2014 [72], Indonesia	418 children aged 0 to 12 years at recruitment	Prospective cohort (3 year follow-up); to investigate growth trajectories relative to nutrition, disease, and hormonal status	Baseline STH prevalence: *A*. *lumbricoides* 30.6%, *T*. *trichiura* 23.4%. STH infection a significant predictor of low height-for-age z-scores in childhood.	Not differentiated by species. Analyses adjusted for sex, age group, and anaemia.
Sayasone et al., 2015 [73], Lao PDR	1313 children, aged 6 months to 12 years	Cross-sectional; to assess and quantify relationship between single and multiple species helminth infection	Parasite prevalence: hookworm 51.0%, *Opisthorchis viverrini* 43.3%, *A*. *lumbricoides* 16.8%, *T*. *trichiura* 10.7%, *S*. *mekongi* 5.6%, *Taenia* spp. 1.2%. Hookworm infection associated with anaemia. Positive association between hookworm infection and pale sub-conjunctiva.	Analyses adjusted for sex, age group, socioeconomic status, nutritional status, and personal hygiene.
**Physical development, fitness, worker productivity—SAC**				
Müller et al., 2011 [74], Côte d’Ivoire	156 SAC aged 7–15 years	Cross-sectional; investigating relationship between schistosomiasis, STH, and physical performance of children	Prevalence: *S*. *haematobium* 85.3%, *Plasmodium* spp. 71.2%, *S*. *mansoni* 53.8%, hookworm 13.5%, *A*. *lumbricoides* 1.3%. Maximum volume of oxygen (VO2 max) influenced by sex and age, but not by hookworm infection or intensity.	Low numbers: 21 hookworm-infected children overall. Analysis adjusted for temperature, relative air humidity, sex, and age.
Degarege and Erko, 2013 [75], Ethiopia	403 SAC aged 5–15 years	Cross-sectional; investigating association between helminth infection and nutritional status of schoolchildren	Prevalence: hookworm 46.9%, *S*. *mansoni* 24.6%, *A*. *lumbricoides* 4.2%, *T*. *trichiura* 1.7%. Hookworm infection associated with low body mass.	Adjusted analysis for age and sex.
Wolde et al., 2015 [76], Ethiopia	450 SAC aged 7–14 years	Cross-sectional; investigating determinants of underweight, stunting, and wasting	Prevalence of helminths (undefined species) 64.3%; *A*. *lumbricoides* 57.6%; hookworm and *T*. *trichiura* prevalence not reported. Ascariasis not associated with being underweight or stunted; reported odds of being stunted increased four times for children who had *T*. *trichiura* infection than children who do not have *T*. *trichiura* infection (but see comment).	Very low numbers—hookworm and *T*. *trichiura* numbers in regression models may be too low to assess anthropometry. Analyses adjusted for sex, age, family size, maternal education, monthly income, household food insecurity, and infection with ascariasis, trichuriasis, or hookworm.
Oliveira et al., 2015 [77], Angola	328 children aged 5–12 years	Cross-sectional; investigating association between helminth infection, anaemia, and stunting	Prevalence of intestinal parasites was 44.2%, comprising *A*. *lumbricoides* (22.0%), *G*. *lamblia* (20.1%), *H*. *nana* (8.8%); hookworms 0.2%. No significant association between *A*. *lumbricoides* infection and anaemia or stunting.	Analyses adjusted for variables P≤0.05 in bivariate analysis.
Casapía et al., 2006 [78], Peru	1,074 SAC aged 8–16 years	Cross-sectional; to determine risk factors for stunting only, and stunting and underweight	STH prevalence 86% (*T*. *trichiura* 77.9%, *A*. *lumbricoides* 60.4%, hookworm 21.3%). Prevalence of either *H*. *nana* or *Enterobius vermicularis* was 10.9%. Hookworm a significant risk factor for stunting and underweight. Moderate and heavy *T*. *trichiura-A*. *lumbricoides* co-infection a significant risk factor for stunting only.	Analyses adjusted for sex and variables of P≤0.1 in univariable analysis.
Verhagen et al., 2013 [79], Venezuela	390 SAC aged 4–16 years from three rural areas	Cross-sectional; to investigate prevalence and associations between intestinal helminth and protozoan infections, malnutrition, and anaemia	Parasite (multiple species) prevalence 68%. Hb levels significantly lower in children with hookworm infection but not *A*. *lumbricoides* infection. Children infected with hookworm had significantly lower weight-for-height/BMI-for-age compared to uninfected children. Children with *A*. *lumbricoides* infection showed reduced weight-for-height/BMI-for-age compared to uninfected children, but only significant for moderate-intensity *A*. *lumbricoides* infection.	Analyses adjusted for age and sex
De Gier et al., 2015 [80], Cuba and Cambodia	1,389 SAC; mean age 8.14 ± 2.07 (Cuba), 2,471 SAC mean age 9.68 ± 2.27 (Cambodia)	Cross-sectional height; to analyse STH and/or zinc associations with height for age z-scores and whether STH and zinc were associated.	STH prevalence 8.4% (Cuba) and 16.8% (Cambodia). In Cuban children, STH infection had strong negative association with height for age.	No species investigations. Analyses adjusted for sex and age.
Yap et al., 2012 [81], China	69 SAC aged 8–15 years	Cross-sectional; to assess feasibility of measuring children’s physical fitness and to relate it to STH infections.	STH prevalence: *T*. *trichiura* 81%, *A*. *lumbricoides* 44%, hookworm 6%. V02 max estimate used within one min during exhaustive exercise of *T*. *trichiura*-infected children lower than that of non-infected children. *T*. *trichiura*-infected children completed fewer 20m shuttle run tests until exhaustion. No significant association between stunting and any STH infection.	Analyses adjusted for age, sex, stunting, and infection status.
Ahmed et al., 2012 [82], Malaysia	289 SAC aged 6–13 years	Pre and post (3 month follow-up after albendazole treatment); to assess risk factors for anaemia and malnutrition, and nutritional impacts of STH infections.	Baseline STH prevalence *T*. *trichiura* 84.6%, *A*. *lumbricoides* 47.6%, hookworm 3.9%. Moderate-to-heavy ascariasis was risk factor for anaemia. Stunting associated with moderate-to-heavy ascariasis and trichuriasis. Post-treatment assessment showed no difference in the mean increments in growth indices between negatively-to-lightly infected and moderately-to-heavily infected groups.	Analyses adjusted for age, gender, mother’s educational level, and household monthly income. *A*. *lumbricoides* not adjusted for *T*. *trichiura* as *T*. *trichiura* was not significant in univariable model. Follow up may not have been long enough to detect changes in anthropometric measurements.
Papier et al., 2014 [83], Philippines	667 SAC aged 10–14 years	Cross-sectional; to investigate whether poor nutrient intake may increase susceptibility to parasitic diseases	Parasite prevalence: *S*. *japonicum* 20.1%, *A*. *lumbricoides* 54.4%, *T*. *trichiura* 71.4%, hookworm 25.3%. Hookworm infection a risk factor for stunting.	Analyses adjusted for variables of P≤0.1 in univariable analysis.
**Physical development, fitness, worker productivity—pregnant women, neonates**				
Ndibazza et al., 2010 [84], Uganda	2,507 pregnant women: (i) 629 albendazole + placebo, (ii) 628 praziquantel + placebo, (iii) 628 albendazole + praziquantel, (iv) 630 placebo + placebo.	Double-blind RCT; 2x2 factorial design: (i) albendazole and placebo, (ii) praziquantel and placebo, (iii) albendazole and praziquantel, (iv) placebo and placebo. Aimed to investigate benefits of deworming during pregnancy on maternal and child health outcomes. Recruited women at antenatal visit (treatment after first trimester); follow-up at delivery and 14 weeks thereafter. Software-generated randomisation. STH diagnosed by Kato-Katz.	2,507 women at baseline; 2,051 (82%) provided post-delivery stool; 1,918 (81%) provided post-delivery blood sample; 1,964 infants (82%) had birthweight recorded; 2,365 infants (99%) were assessed for congenital anomalies at birth. Baseline parasite prevalence 68% (hookworm 45%, *S*. *mansoni* 18%). At delivery no overall effect of albendazole on maternal anaemia, but suggestion of benefit of albendazole among women with moderate to heavy hookworm. No effect of albendazole on mean birth weight or on proportion of low birth weight. Anthelmintic use during pregnancy showed no effect on perinatal mortality or congenital anomalies.	Sample size calculations based on different trial outcomes. Study powered to detect 0.3g/L difference in maternal Hb and 70g difference in infant birthweight for either intervention [84]. Adequate power to detect whole group effects but not perinatal mortality effects in subgroup analyses [84]. Excluded women with clinical requirements to treat low Hb; may have excluded those most likely to benefit from interventions [84]. Loss to follow-up did not vary between trial arms. Analyses adjusted for albendazole or praziquantel (according to treatment being investigated in the model).
van Eijk et al., 2009 [85], Kenya	390 pregnant women	Cross-sectional; to investigate STH risk factors and effects among pregnant women	STH prevalence: 76.2% (*A*. *lumbricoides* 52.3%, hookworm 39.5%, *T*. *trichiura* 29.0%). STH infections not associated with clinical symptoms or low body mass index. Hookworm infection associated with a lower mid upper arm circumference.	Models adjusted for malaria, marital status, treatment of water and a report of soil eating, and other STH.
Fairley et al., 2013 [86], Kenya	696 pregnant women	Cross-sectional; to investigate associations of maternal helminth infection and malaria-helminth co-infection on birth outcomes	Prevalence in mothers: *P*. *falciparum* 42.7%, *S*. *haematobium* 30.6%, filariasis 36.2%, hookworm 31.5%, *T*. *trichiura* 5.9%. Hookworm or trichuriasis infection not associated with neonatal low birth weight or a low weight z-score.	Model adjusted for maternal age, socioeconomic status, education level, marital status, gravida, and area of residence.
Aderoba et al., 2015 [87], Nigeria	178 women with singleton pregnancy	Cross-sectional; investigating STH prevalence during pregnancy and associated adverse maternal and infant outcomes	STH prevalence 17.4% (8.4% *A*. *lumbricoides*, 4.5% *T*. *trichiura*, 14.0% hookworm). Mean Hb concentration lower among STH infected women than those without. Mild anaemia more common among STH infected women. Mean birth weight lower for infants of STH-infected women; prevalence of low birth weight higher in this group.	Very low numbers (31 women infected); no species investigations. Analysis adjusted for maternal weight, parity, social class, HIV status, and pregnancy duration at booking.
Boel et al., 2010 [88], Thai-Burmese border	339 pregnant women (in 1996), 490 (in 2007)	2 cross-sectional studies (1996 and 2007); to assess relationship between STH infection and the progress and outcome of pregnancy	Overall STH prevalence 70% (hookworm 43%, *A*. *lumbricoides* 34%, *T*. *trichiura* 31%). Hookworm infection associated with an increased risk of anaemia. Hookworm associated with low birth weight.	Adjusted models used variables significantly associated in univariate analysis, including malaria, primigravid/multigravid status, and participation in the 1996 survey.
**Physical development, fitness, worker productivity—all ages**				
Baird et al., 2011 [89], Kenya	Original cohort were approx. 30,000 SAC from 75 schools [90]	Longitudinal economic analysis, examined impact of deworming programme on adult living standards by following participants from deworming program that began in 1998.	Followed up 83% of deworming programme participants over a decade. Self-reported health, years enrolled in school, and test scores improved significantly, and hours worked increased by 12% in the treatment group. Treated individuals reported eating an average of 0.1 additional meals per day. Within the subsample working for wages, earnings were >20% higher for the treated group. Most of earnings gains are explained by sectoral shifts, including doubling of manufacturing employment. Small business performance improved among the self-employed. A lower bound on the annualized social internal rate of return to deworming is large, at 83%.	Unclear if this has been independently peer reviewed.
Degarege et al., 2013 [91], Ethiopia	480 febrile outpatients aged 1–80 years; mean age (SD) 23.1 (14.2) years	Cross-sectional; to investigate associations between helminth infections and ABO blood group, anaemia, and undernutrition	Parasite prevalence 50.2% (*A*. *lumbricoides* 32.7%, *T*. *trichiura* 12.7%, *S*. *mansoni* 11.9%, hookworm 11.0%). Individuals infected with only *T*. *trichiura* had significantly lower mean haemoglobin level compared to uninfected individuals. Odds of developing anaemia higher in individuals infected only with hookworm compared to uninfected individuals. Increased intensity of *A*. *lumbricoides* infection significantly associated with decrease in haemoglobin level and with increase in anaemia prevalence. Odds of being underweight significantly higher in *A*. *lumbricoides*-infected individuals aged ≤5 and *T*. *trichiura*-infected individuals aged ≥20 years, compared to uninfected individuals.	Analyses adjusted for age, sex, and nutritional status.
**Cognitive development**^#^				
Ebenezer et al., 2013 [92], Sri Lanka	1,190 SAC (615 intervention, 575 control)	Unblinded cluster RCT (grade 4 in schools); 49 schools received deworming and weekly iron supplementation for 6 months (intervention), 49 schools received placebos for both anthelmintic and iron (control); follow-up 6 months. Aimed to assess impact of school-based deworming and iron supplementation on individual cognitive abilities. Software-generated randomization. STH diagnosed by Kato-Katz.	Baseline enrolment of 1,621 children; 1,190 children (73.5%) included in analyses. Baseline STH prevalence 25.7% (*A*. *lumbricoides* 21.0%, *T*. *trichiura* 6.1%, hookworm 5.3%). No impact of deworming and iron supplementation found on Hb levels, anaemia, cognitive test, or educational test scores.	Analyses adjusted for age, sex, baseline nutritional status, individual socioeconomic indicators (parental education), school-level indicators (whether or not school had an ongoing midday meal programme), interaction between treatment and low nutritional status, and high-intensity worm burden. Hb level and anaemia prevalence differed between treatment and control groups (adjusted in models). Loss to follow-up of two control schools, but similar loss to follow-up overall across treatment and control arms [92].
Thériault et al., 2014 [93], Peru	1,088 SAC, mean age 10.3 ± 1.1 years (517 intervention, 571 control for analyses)	Cluster RCT (nine intervention schools, nine control schools); both trial arms received deworming treatment; intervention arm received 4 months of health hygiene education aimed at increasing knowledge of STH prevention; follow-up 4 months. Aimed to measure impact of health hygiene education on absenteeism. Software-generated randomization. STH diagnosed by Kato-Katz.	1,486 students at baseline; 1,088 (73.2%) analysed. Baseline STH prevalence 72.1% in intervention schools, 78.1% in control schools. At completion, overall absenteeism rates at intervention and control schools not significantly different. Post-trial non-randomized analyses showed students with moderate-to-heavy *A*. *lumbricoides* infections and light hookworm infections 4 months after deworming had, respectively, missed 2.4% and 4.6% more schooldays during follow-up period than uninfected counterparts.	Data from [94]. Analyses adjusted for socioeconomic status and age. 4 months may not have been long enough to detect changes in absenteeism from health education intervention. Infection status between control and intervention groups differed; may have affected estimates. Study powered to detect a different outcome, therefore analyses may be underpowered [93]. Attendance measured by teacher logs rather than direct observation (systematic bias affecting both trial arms) [93].
Ahmed et al., 2012 [95], Malaysia	289 SAC aged 6–13 years (same cohort as reported in [82])	Pre and post (3 month follow-up after albendazole treatment); to determine possible relationship between intestinal helminthiasis and school absenteeism	Baseline STH prevalence: *T*. *trichiura* 84.6%, *A*. *lumbricoides* 47.6%, hookworm 3.9%. Infection of moderate-to-heavy worm burdens and anaemia identified as significant risk factors of high absenteeism among the subjects. Following treatment of infected children, school absenteeism reduced significantly by about 16% among the pupils.	Multivariable models included ascariasis, trichuriasis, anaemia status, stunting, underweight, mother’s employment status, mother’s education, and father’s education. Multivariable results a bit unclear, particularly how models were built and whether consideration was given to anaemia, stunting, and underweight as potential effect modifiers. Given this, and short follow-up, very interesting association between STH infection and absenteeism.
Ezeamama et al., 2012 [96], Philippines	253 *S*. *japonicum*-infected SAC aged 7–19 years	Prospective cohort study followed for 18 months; to determine whether treatment of intestinal parasitic infections improves cognitive function. Separately assessed changes in cognitive test scores for: (i) treatment-related *S*. *japonicum* intensity decline, (ii) spontaneous reduction of single STH species, and (iii) ≥2 STH infections among *S*. *japonicum*-infected children.	At baseline, 97% concurrent infection with *S*. *japonicum* and ≥1 STH species. Baseline prevalence of *A*. *lumbricoides* 79.9%, *T*. *trichiura* 95.6%, hookworm 50.6%. At follow-up, a decline versus no change/increase of any individual STH species and joint decline of ≥2 STH species associated with higher scores in Wide Range Assessment of Learning and Memory (WRAML) learning test. Hookworm and *T*. *trichiura* declines independently associated with improvements in WRAML memory scores as was the joint decline in ≥2 STH species. Baseline coinfection by ≥2 STH species associated with low Philippine nonverbal intelligence test (PNIT) scores.	Adjusted analyses by age, sex, and socioeconomic status. Considered potential effect modification from helminth infection intensity, underweight, and anaemia.
Liu et al., 2015 [97], China	2179 SAC aged 9–11 years	Cross-sectional; to examine relationship between STH infections and developmental outcomes	STH prevalence: 42% (*A*. *lumbricoides* 31%, *T*. *trichiura* 22%, hookworm 1%). Infection with ≥1 STH associated with worse cognitive ability, worse nutritional status, and worse school performance than no infection. Children with *T*. *trichiura* infection, either single infection or co-infected with *A*. *lumbricoides*, experienced worse cognitive, nutritional, and schooling outcomes than uninfected peers or children infected with only *A*. *lumbricoides*.	Analyses adjusted for gender, age, boarding status, ethnicity, ever eats uncooked meat / vegetables, ever drinks unboiled water, and socioeconomic characteristics
Additional *A*. *lumbricoides* morbidity				
Alam et al., 2010 [98], Bangladesh	138 consecutive cases of biliary and pancreatic ascariasis (BPA) in adults, mean age 36.8 ± 16.1 years	Case series	Comparison of clinical BPA morbidity from dead and living *A*. *lumbricoides* worms. 98 patients had living worms, 40 had dead worms. Males were more prone to develop dead worm BPA. Presentations for biliary colic (131; 94.9%), acute cholangitis (30; 21.7%), obstructive jaundice (19; 13.8%), choledocholithiasis (20; 14.5%), acute pancreatitis (10; 7.2%), acute cholecystitis (6; 4.3%), liver abscess (2; 1.4%), hepatolithiasis (3; 2.2%), stricture of common bile duct (2; 1.4%), pancreatic abscess (1; 0.7%), and cirrhosis of liver (1; 0.7%). Surgical intervention required in five patients. Recurrences of stone and cholangitis occurred only in those with dead worms. Biliary ascariasis with dead worms deemed more dangerous than that with living worms.	
Baba et al., 2009 [99], India	207 patients admitted with diagnosis of intestinal obstruction aged 3–14 years	Case series	131 patients diagnosed as having obstruction due to ascariasis. Most patients 3–5 years of age. 64 patients needed operative intervention of either enterotomy, milking of worms or resection anastomosis. Appendicular perforation was seen in four and worm in gall bladder in one patient. Surgical complications were wound infection in 17, burst abdomen in four and faecal fistula in three patients.	
Mukhopadhyay, 2009 [100], India	42 cases of hepatobiliary ascariasis, adults aged between 20–50 years	Case series	Most common presentation was upper abdominal pain in 95.2% of the patients (40 patients). Complications included obstructive jaundice in 28.6% (12 patients), cholangitis in 16.7% (seven patients), acute pancreatitis in 2.4% (one patient), and hepatic abscess in 2.4% (one patient). History of worm emesis present in 38.1% (16 patients). History of previous cholecystectomy present in 16.7% (seven patients) and endoscopic sphincterotomy in 4.8% (two patients). Conservative management successful in 83.3% (35 patients). During follow-up, worm reinvasion of biliary system occurred in 7.1% (three patients).	
Additional *T*. *trichiura* morbidity				
Khuroo et al., 2010 [101], India	Ten cases of TDS in adults	Case series	No patients had growth retardation, malnutrition, or immunodeficiency. Abdominal symptoms in one patient; nine had no abdominal symptoms. Large numbers of actively motile *T*. *trichiura* in right colon (seven patients), ileum (one), left colon (one), and whole colonic mucosa (one). Mucosal changes included petechial lesions, blotchy mucosal haemorrhages, and active mucosal oozing.	TDS has not previously been described in adults.
Kaminsky et al., 2015 [102], Honduras	Children aged 12 years or younger	Hospital-based study; severe trichuriasis cases identified by routine stool examination from hospitalised patients.	11,528 faecal samples examined between March 2010 and September 2012; of these, 122 (1.0%) *T*. *trichiura* infections were diagnosed. Morbidity included dysentery of several months’ duration, severe anaemia, and stunting. Heavy *T*. *trichiura* infections included egg counts from 232 to 3,520 eggs, Hb 3.4 to 10.8 g/dL, eosinophilia, severe malnutrition, and growth stunting. Orally administered drugs prescribed at different dosages and duration; no cure or egg excretion control was exercised before patient release. A range of 340 to about 10,000 worms were recovered after treatment from eight patients.	Cases selected on basis of high *T*. *trichiura* egg counts. Descriptive analysis; some morbidity data (e.g., persistence of dysentery) self-reported. There is a need to conduct detailed community studies in trichuriasis morbidity, effective treatment assessment, and clinical response in severely malnourished parasitised children [102].

* RCTs are focus given prior systematic reviews, however one cross-sectional study for *T*. *trichiura* is included.

^#^ Two replication analyses [5,6] are not included in this table as they were analysed in [4].

We looked at coinfections among STH species. For STH and non-STH parasite interactions, STH and vaccine interactions, and associations with allergies, atopy, and asthma, we note that effects on morbidity are likely to be synergistic and that research is under way to investigate these effects. However, discussion of these is beyond the scope of the current review.

After applying the above search criteria, studies were further excluded from Table 3 if they had been analysed in one of the systematic reviews, if they did not report results for STH species separate from other parasites, or results on haemoglobin, anaemia, or a physical or cognitive measure (with the exception of the case series for *A*. *lumbricoides* and *T*. *trichiura*). For this reason, studies that reported prevalence or intensity of infection only (indirect morbidity measures), or changes in these following interventions, were not reported. Furthermore, this means that for some studies, not all study outcomes were included in our evidence tables. Critical appraisal checklists were again followed to assess these studies [14].

## Findings from Observational and Experimental Evidence

Tables 1 and 2 provide a summary of evidence for STH morbidity derived from previous narrative and systematic reviews. These have been used to determine current knowledge and evidence gaps. Table 3 summarises the information for recent observational and experimental evidence, including 44 studies by key topic area. Whilst some general statistical and epidemiological comments have been provided on study design, processes, analyses, and limitations, we have not applied formal scoring criteria to these studies.

### STH entry and establishment within the human host

No recent epidemiological studies addressing STH entry and establishment in the human host were identified. Sequelae (Table 1) associated with STH entry into the human host tend to be regarded as transient events, often reported as features of the STH life cycle rather than in terms of quantifiable morbidity on the host. Narrative reviews have reported a broad range of symptoms following larval migration to, and establishment within, the gastrointestinal tract (Table 1). The only epidemiological studies likely to be ethically acceptable to quantify these symptoms would be cohort studies being conducted on people moving to endemic areas for the first time, or on very young children being exposed to STH for the first time. Quantifying infection-related or gastrointestinal-related morbidity from STH does not seem to be a current research focus.

### Blood loss, haemoglobin, and anaemia

The evidence for negative impact of STH infections is strongest for morbidity due to blood loss and anaemia. There is clear evidence that hookworms in particular cause blood loss from feeding on mucosal tissue in the small intestine [23], thereby causing iron-deficiency anaemia. However, anaemia is of multifactorial origin [103], and it is difficult to disentangle the effect from all other potential confounders. Furthermore, there are likely to be different impacts of anaemia in different age groups. With the exception of two systematic reviews investigating impact in pregnant women [52,53] and one that investigated observational evidence separately for school-aged children and adults [12], systematic reviews have not investigated age-dependent effects. Whilst this is likely due to lack of evidence, this is particularly important for anaemia indicators.

Evidence from systematic reviews and recent experimental studies is mixed; not all studies have found an impact of hookworm (or STH more generally) on either haemoglobin levels or anaemia. Three systematic reviews (one in non-pregnant populations, one in pregnant populations, and one across populations), confirm previous observational direct correlations between intensity of hookworm infection and reduced haemoglobin, or improvements in haemoglobin levels following deworming [12,13,53]. Two other systematic reviews (one in children aged 0 to 16 years, one in pregnant women) [4,52] found no evidence for reduced anaemia following deworming. Two RCTs found changes in STH intensity or haemoglobin levels, but not in levels of anaemia, following deworming [55,58]. This is possibly due to differing levels of hookworm burden [104] and hookworm species, different anthelmintics used, underlying nutritional status, or other confounding factors, as well as specific differences in systematic reviews as discussed below.

It is only since 2002 that deworming has been advocated during pregnancy (after the first trimester), with the result that experimental data on maternal and child outcomes are relatively recent and more evidence is needed. Whilst data are somewhat dated, iron-deficiency anaemia has been suggested to be responsible for 20% of maternal deaths worldwide [23], with estimates that hookworm causes at least 30% of moderate or severe cases of anaemia among this population group [105]. The WHO estimates that more than half of pregnant women in developing countries have morbidity related to iron-deficiency anaemia [27]. Given this high prevalence, quantitative investigations into the role of hookworm in anaemia-related maternal mortality are required. No estimates of hookworm-associated mortality are currently included in GBD calculations.

Studies vary with regards the involvement of *T*. *trichiura* in blood loss and anaemia. *T*. *trichiura*-associated blood loss has been previously inferred to only become significant in heavy infections [48]; newer evidence [59] is in agreement with this. Interestingly, in one recent study undertaken in a high *T*. *trichiura* and *A*. *lumbricoides* (but low hookworm) endemic environment, moderate to heavy *A*. *lumbricoides* infection was a risk factor for anaemia in school-aged children [82]. Further experimental evidence investigating the impact of STH on haemoglobin and anaemia, particularly in preschool-aged children and pregnant women, is required.

### Physical development, fitness, and worker productivity

Whilst this is the focus area for which we found the greatest quantity of recent studies, results for impact of STH on measures of height, weight, and head circumference are mainly observational and vary widely. Some studies found impacts of *T*. *trichiura*, *A*. *lumbricoides*, and/or hookworm on height but not weight, weight but not height, both height and weight, height and head circumference, and some studies found no impacts at all (Table 3). Underlying prevalence and infection intensity varied considerably by geographic location and, although most studies adjusted for some potential confounders, it is difficult to control for the role of malnutrition within populations, which could have had a major influence on results. Generally, more studies reported associations with stunting and wasting than studies that did not. The Cochrane systematic review found that deworming may increase weight gain [4], but was inconclusive on other physical health measures.

There are inherent difficulties in detecting growth changes in school-aged children [106], with, amongst other things, an appropriate length of follow-up time required to adequately assess these. However RCTs need to balance sufficient follow-up to detect effects with the potential for STH re-infection. Given rapid growth and potentially greater STH morbidity (evidenced by intensity of infection data) in preschool-aged children and the fact that, in some areas, preschool-aged children are now included in deworming programmes, greater emphasis on investigating morbidity in this cohort is required. It could be that this age group is where the strongest evidence of effect may lie.

It is biologically plausible that young girls who grow poorly become stunted women with a greater chance of giving birth to low birth weight infants who are likely to be stunted in adulthood [37]. Whilst longitudinal investigations into the impact of chronic STH infection in girls, following their general health status as they become mothers, have not been conducted, studies investigating pregnancy outcomes are being conducted. In terms of albendazole impact on pregnant women and neonates, an RCT not included in the systematic reviews provides further evidence of the lack of clear benefit of albendazole on maternal or neonatal health [84]. Some observational studies (but again, not all) [88] reported associations between hookworm infection and low birth weight.

We found one recent attempt in the published literature to investigate effects of STH on longer-term schooling and worker productivity; this analysis found improved economic outcomes for a cohort of dewormed individuals followed over approximately 10 years [89], similar to a previous economic analysis [107]. There is negligible other direct evidence that STH infections reduce adult productivity. However, the health consequences, such as anaemia, are known to affect productivity, and hookworm could be a major contributor to this. More well-designed longitudinal analyses are needed.

### STH impact on cognitive development, school performance, and absenteeism

The impact of STH on cognitive development is the area that has come under greatest scrutiny over the years. It is extremely complex to measure accurately. Cognitive psychology is a dynamic field, encompassing different theories of the interplay of a broad range of psychological and environmental factors. Impaired cognition is rarely from a single cause, with an array of cognitive tests needed to assess impacts such as STH on a range of cognitive functions [106]. It is perhaps not surprising that despite much research undertaken to investigate whether STH contribute to cognitive impairment in children, few conclusions have been able to be drawn. The Cochrane systematic review investigated three RCTs undertaken in children of known high-intensity infection status, specifically designed to measure cognitive outcomes, but did not meta-analyse these due to different outcome definitions and therefore drew no new conclusions. Recent experimental and observational studies further illustrate this. Firm evidence continues to be elusive. Recent evidence investigating school absenteeism is sparse.

### Additional clinical morbidity from STH species

Severe clinical complications such as trichuris dysentery syndrome (TDS), or intestinal obstruction and hepatopancreatitis as a result of *A*. *lumbricoides* infections, are relatively rare and represent only a small portion of the disease burden [108], although they are sufficiently serious to warrant attention. Apart from very few case series, there is an almost complete lack of epidemiological investigation into quantifying these acute complications over recent years. This is important to highlight, as ascariasis is the most common of the STH infections and causes the majority of STH mortality [109]. In the absence of recent information, it is unclear how mortality estimates have been derived [110]. It is additionally unclear whether any recent estimates exist for one of the most serious presentations of ascariasis, hepatopancreatic ascariasis (HPA), or a disease known as recurrent pyogenic cholangitis (RPC) (caused by stone formation, usually around dead *A*. *lumbricoides* in the bile duct). This has been epidemiologically linked to recurrent biliary invasion by *A*. *lumbricoides* in endemic areas [111]. Similarly, there appear to be no detailed quantitative investigations of trichuriasis, particularly TDS, in recent years. TDS can cause major, acute disease that is sometimes life-threatening [28]. There are no recent empirical data on either global incidence of TDS or any attributable mortality.

Current STH prevalence and burden estimates do not include *Ancylostoma ceylanicum*, which recent data show to be the second most prevalent hookworm species after *N*. *americanus*, in some Asian countries [50]. Whilst evidence is scant, *A*. *ceylanicum* may have more severe morbidity than *A*. *duodenale* [112,113]. Studies of *A*. *ceylanicum*-associated morbidity are somewhat dated and highlight the evidence gap arising from a failure to investigate morbidity associations. Diagnosis of *A*. *ceylanicum* requires coprodiagnostic molecular biology techniques not readily available in developing countries; however, in the current era of large-scale deworming programmes, there is growing impetus for utilisation of contemporary diagnostic methods, hence renewed focus on this hookworm species is needed.

It is clear that many morbidities associated with STH cannot be investigated in an experimental design and, further, that many associations (such as maternal mortality from hookworm) are not feasible to investigate at all. In assessing evidence for deworming on STH-associated morbidities, the Cochrane Collaboration has exclusively considered RCT and quasi-RCT evidence in its systematic reviews of deworming. As has been raised elsewhere [7,8], this major limitation results in lack of consideration of a vast quantity of broader evidence of association. The Cochrane systematic review is assessed in detail below.

## A Critical Appraisal of a Cochrane Systematic Review

Cochrane systematic reviews are regarded as the benchmark for high-quality evidence, utilising rigorous methodologies undertaken in accordance with a set protocol. In the most recent Cochrane review [4] on the health benefits of deworming, the authors considered length of trial follow-up and different assessment points. They undertook analyses by classes of STH infection intensity. They further investigated dose number as either single dose or multiple dose treatments. They were not able to undertake analyses by age group due to insufficient data. The authors considered only absolute measures of heights and weights. As is appropriate, trials were not pooled where the outcome definitions varied, such as cognitive tests.

Under the rules of the Cochrane Collaboration, the systematic review protocol must be produced prior to undertaking the review [114]. It is clear from the report that a protocol was developed. However, the protocol is not publicly available on the Cochrane website and we could not verify whether key points raised below were written in the protocol, or whether changes were made during the review process, possibly due to lack of data. The research objective of the Cochrane systematic review was “to summarise the effects of giving deworming drugs to children to treat soil-transmitted intestinal worms, in weight, haemoglobin, and cognition; and the evidence of impact on physical well-being, school attendance, school performance, and mortality” [4]. We believe this is a situation in which the null hypothesis and the intended subgroup analyses need to be clearly stated and be verifiable with the protocol. One interpretation of the null hypothesis from the stated objective is that “deworming does not improve the listed health outcomes.” This is supported by the description of the participants as “infected children identified by screening in community trials. All children must have lived in endemic areas” [4]. This does not clearly imply consideration of unscreened children. Yet, for data synthesis, the participants are separately analysed according to these two groups: infected children and all children living in an endemic area. Thirty-seven of the 45 included RCTs were based on mass drug distribution of an unscreened population [4]. Therefore, the alternative interpretation of the null hypothesis also follows: “mass drug distribution as delivered to all children in endemic areas does not improve health outcomes.” The objective, participants, and intended subgroup analyses need to be more clearly explained, as the two hypotheses require a major conceptual shift in interpretation and raise different questions in terms of included studies and how outcomes are determined. The issue of considering unscreened children has already been strongly criticised, primarily because international policy promotes treating all children in endemic communities, and a systematic review is not required to establish that treating uninfected children will have no health benefit on these children [7,8].

With the analysis of unscreened children, trials have been pooled irrespective of STH species, treatment types, and drug distribution strategies. Conceptually, pooling trials in systematic reviews is appropriate; however, for STH there is a very high level of heterogeneity, and this approach is prone to methodological flaws. By considering RCTs conducted in different locations in which screening has not been done, there is no baseline assessment of STH prevalence and/or intensity. The underlying assumption is that baseline prevalence is the same between intervention and control groups, which enables post-intervention assessment attributable to the intervention (provided randomisation adequately enabled control for confounding and that there was no systematic differential bias between groups). Whilst justifiable for RCTs, this causes a difficulty for systematic review methods, as there is marked STH heterogeneity in different endemic areas and, if baseline testing is not done, there must be another estimate to determine the STH of greatest prevalence in the population (e.g., from other epidemiological surveys) to ensure that heterogeneity is addressed when pooling RCTs. In the systematic review, no baseline of STH prevalences are reported. Whilst evidence is limited, there is sufficient prior knowledge of differential impacts of STH on morbidity outcomes, e.g., the role of hookworms, but not *A*. *lumbricoides*, in blood loss, to indicate that pooling of STH is not an accurate way of assessing morbidity. Similarly, it is also not accurate to pool different anthelmintic treatments of known and well reported [115,116] very different efficacies according to STH. Lastly, drug distribution strategies, particularly targeted delivery to school-aged children versus mass drug administration to all community members [117], is currently a major area of research investigation; differential impacts between school and non-school child cohorts are likely according to delivery strategies of targeted school programmes versus broader community treatments [118], or different frequency treatments as are recommended according to endemicity [117]. Such pooling of studies would cause dilution of effects due to different STH responsiveness to treatments with known differential efficacies, potentially delivered according to different strategies. Thus, the Cochrane authors appear to have not pooled like with like. It comes as no surprise that few conclusions can be drawn from the Cochrane systematic review.

The rationale for pooling studies may have been data-driven or aimed at replicating the deworming programme context. A more robust approach would be to apply the method of Smith and Brooker [12], who used known baseline prevalence of hookworm infection, analysed by hookworms only, and by anthelmintic drug classes separately. It is very probable indeed that there is insufficient evidence to undertake meta-analyses for many morbidity outcomes by different STH and different anthelmintics. However, this is not a valid rationale for pooling them. If such meta-analyses cannot currently be done, there is insufficient evidence to say that deworming does not contribute to health outcomes. As has been indicated by others [8], this is not the same as a lack of effect.

The lack of evidence is correctly and clearly pointed out by the Cochrane authors with regards treatment of children with known STH infection. Participant and trial numbers for many outcomes were very low and many of the results were not sufficient to meta-analyse. Lack of sufficient data was also the reason why subgroup analysis by age was not conducted. This is, however, an extremely important evidence gap given differing age prevalence profiles of STH and the likely greater morbidity in preschool-aged children (with the consequence that these effects, too, could be diluted across age groups). The authors conclude that most evidence is of very low, low, or moderate quality. The most useful and important conclusion of the systematic review is that there is a major shortfall in evidence for most morbidities to feed into meta-analyses in the first place. Until such time as evidence is generated, meta-analyses will not be able to appropriately assess the health benefits of STH interventions.

If the protocol was publicly available, we could specifically ascertain whether the included trials met the protocol, whether excluded trials did not meet the protocol, and whether other trials should have been identified under the terms of the published search strategy. Other authors have noted omission of RCTs that ideally should have been considered [8]. Further evidence that has not been provided in the most recent Cochrane systematic review include GBD estimates since 2003 and a 2010 systematic review that found statistically significant effects for some anthelmintics on haemoglobin [12]. Finally, the authors have raised the issue of young children choking on deworming tablets in their discussion, referring to an unreferenced WHO newsletter. Their viewpoint is not derived from their systematic analyses.

## Conclusion

In this manuscript, we have found that there is a paucity of recently collected data to inform our knowledge of STH morbidity. In particular, relatively little quantifiable evidence of STH morbidity has been forthcoming in recent years. Of the evidence that has been provided, few firm conclusions can be drawn. Perhaps this, too, is a reflection of the insidiousness of STH. Alternatively, this could be partly a result of trials that are powered to measure different primary outcomes; secondary morbidity outcomes may therefore be inadequately powered for effects to be detectable. Furthermore, this may be because intervention trials are impossible to conduct over sufficient time periods to assess deworming impacts on morbidity in the manner that such programmes are delivered in real-world settings (i.e., repeated rounds administered throughout childhood). There is also a possibility that our review has applied selection criteria that could have excluded key evidence. We considered that applying these criteria was the most accurate way to differentiate between direct and indirect STH morbidity measures. The main discrepancy that the Cochrane systematic review highlights is insufficient and heterogeneous underlying evidence. This is reinforced by our own findings.

We do have an evidence problem regarding STH morbidity and health effects of deworming. The use of prevalence and, to a lesser extent, intensity of infection as indicators for intervention planning, monitoring, and evaluation may have reduced the impetus to investigate more direct morbidity measures. As a consequence, we might not currently be able to prove the benefits of deworming. Furthermore, evidence of morbidity may become increasingly hard to detect over time if prevalence and intensity continue to reduce in populations. This is obviously a good outcome, but a poor basis upon which to make any assessments. Our main conclusion is that further investments in appropriately designed studies that are powered to measure changes in direct STH morbidity indicators are urgently required.

**Key Learning Points**

- There is a paucity of quantifiable evidence of STH morbidity in recent years when assessed by direct morbidity measures such as changes in height, weight, haemoglobin, and cognition.
- The most recent Cochrane systematic review has assessed possible benefits of deworming on morbidity outcomes by pooling RCTs of deworming regardless of individual infection status, STH species, type of anthelmintic drug, and distribution strategy. In our opinion, this is methodologically inaccurate given current knowledge of STH heterogeneity. There may be insufficient evidence to prove benefits of deworming, but this is not the same as the authors’ conclusion of lack of an effect.
- Careful consideration needs to be given to use of systematic reviews of RCTs for measuring improvements in morbidity from deworming. Furthermore, there needs to be clarification of the role of observational evidence for assessing STH-associated morbidity given that not all morbidity investigations are feasible within an RCT design.
- Studies designed to detect direct morbidity from STH are urgently required to strengthen evidence for deworming.

**Top Five Papers**

1 Taylor-Robinson DC, Maayan N, Soares-Weiser K, Donegan S, Garner P. Deworming drugs for soil-transmitted intestinal worms in children: effects on nutritional indicators, haemoglobin, and school performance. Cochrane Database Syst Rev. 2015;7: CD000371.
2 Smith JL, Brooker S. Impact of hookworm infection and deworming on anaemia in non-pregnant populations: a systematic review. Trop Med Int Health. 2010;15(7): 776–95.
3 Gulani A, Nagpal J, Osmond C, Sachdev HP. Effect of administration of intestinal anthelmintic drugs on haemoglobin: systematic review of randomised controlled trials. BMJ. 2007;334(7603): 1095.
4 Brooker S, Hotez PJ, Bundy DA. Hookworm-related anaemia among pregnant women: a systematic review. PLoS Negl Trop Dis. 2008;2(9): e291.
5 Haider BA, Humayun Q, Bhutta ZA. Effect of administration of antihelminthics for soil transmitted helminths during pregnancy. Cochrane Database Syst Rev. 2009(2): CD005547
